# A Rare Case of Placenta Increta at Uterine Fundus

**DOI:** 10.7759/cureus.67147

**Published:** 2024-08-18

**Authors:** Paidi Naga Rachana, Bharathna Chennuru, Sukesh Kathpalia, Shilpa Kshirsagar

**Affiliations:** 1 Obstetrics and Gynaecology, Dr. D. Y. Patil Medical College, Hospital and Research Centre, Dr. D. Y. Patil Vidyapeeth (Deemed to be University), Pune, IND

**Keywords:** high-risk pregnancy, morbid adherent placenta, obstetric hysterectomy, ceserean section, placenta accreta syndrome

## Abstract

Adherent placenta means a placenta that is not delivered spontaneously or even after manual removal within 30 minutes of baby birth. It is an uncommon and frequently unanticipated event with serious potential health circumstances and it should be managed by the medical team. This case study presents a rare instance of placenta increta in a 25-year-old woman, second gravida, at 36 weeks of gestation, with a history of cesarean section 16 months prior due to chorioamnionitis. The patient presented to the labor room in active labor, and antenatal ultrasound indicated placental implantation on the posterior surface of the upper uterine segment. Given the short inter-delivery interval, an emergency preterm lower segment cesarean section (LSCS) was performed, resulting in the birth of a healthy baby girl weighing 1.8 kg. During surgery, a morbidly adherent placenta was found over the fundus of the uterus. Following consultations with the patient and her relatives, an emergency obstetric total hysterectomy was performed. Intraoperatively, the patient received one unit of packed cell volume (PCV) and, postoperatively, two additional units of PCV and two units of fresh frozen plasma (FFP) were administered. On the third postoperative day, the patient developed right lung consolidation, necessitating a five-day stay in the Obstetric Intensive Care Unit (OBICU). The remaining postoperative period was uneventful, and the patient was discharged on the 10th postoperative day with the healthy infant.

Placenta accreta, including its variants increta and percreta, represents abnormal placental implantation into the uterine wall, a condition whose incidence is rising due to increased cesarean sections and improved imaging detection.

## Introduction

Placenta accreta spectrum (PAS) disorders, encompassing placenta accreta, increta, and percreta, are characterized by the abnormal adherence of the placenta to the uterine wall, with varying degrees of invasion into the myometrium. Placenta accreta involves the superficial attachment of the placenta to the myometrium without intervening decidua. Placenta increta denotes deeper invasion into the myometrium, while placenta percreta penetrates through the myometrium and may invade adjacent organs, such as the bladder [1]. The incidence of PAS disorders has been on the rise, primarily due to an increasing number of women undergoing multiple cesarean sections and the enhanced ability to detect these conditions through advanced imaging techniques [2,3].

PAS disorders pose significant risks to both maternal and fetal health due to the high potential for massive obstetric hemorrhage, the need for blood transfusions, and the increased likelihood of hysterectomy [4]. Furthermore, PAS can result in preterm delivery and associated neonatal complications. The increasing prevalence of PAS disorders necessitates heightened awareness and improved diagnostic and management strategies to mitigate these risks [5].

Several risk factors contribute to the development of PAS disorders. The most significant risk factor is a history of cesarean delivery. The risk of PAS increases with the number of prior cesarean sections, with a reported incidence of up to 67% in women with placenta previa and four or more cesarean sections [6]. Other risk factors include placenta previa, prior uterine surgery (such as dilation and curettage, manual removal of the placenta, synechiolysis, or myomectomy), increasing maternal age, high parity, and assisted reproductive technologies [7]. The presence of these risk factors necessitates careful antenatal monitoring and consideration of PAS in the differential diagnosis.

The diagnosis of PAS disorders is challenging and often not confirmed until delivery when attempts at manual removal of the placenta fail. However, antenatal diagnosis is crucial for planning appropriate management to reduce maternal and fetal morbidity and mortality. Ultrasound, particularly transvaginal ultrasound (TVS), is the primary imaging modality used for the diagnosis of PAS. Key ultrasound findings include the presence of placental lacunae, loss of the retroplacental clear space, and abnormal color Doppler patterns indicating increased vascularity at the placental-myometrial interface [8]. Magnetic resonance imaging (MRI) can provide additional information, especially in cases where ultrasound findings are inconclusive. MRI features indicative of PAS include uterine bulging, heterogeneous signal intensity within the placenta, and dark intra-placental bands on T2-weighted imaging [9].

Histopathological examination remains the gold standard for confirming PAS, revealing features such as partial or total absence of the decidua basalis and imperfect development of the fibrinoid layer (Nitabuch’s layer) [10]. Elevated levels of alpha-fetoprotein (AFP) have also been observed in PAS cases, although this is not a definitive diagnostic marker [11]. The cornerstone of management is timely cesarean hysterectomy, which entails delivery of the infant followed by removal of the uterus with the placenta in situ to prevent hemorrhage. Preoperative preparation includes patient counseling and consent for arterial catheterization to occlude pelvic blood flow and reduce intraoperative bleeding [12]. Intraoperative and postoperative management focuses on controlling hemorrhage and supporting maternal hemodynamics through transfusions and critical care support as needed [13].

In conclusion, the rising incidence of PAS disorders underscores the importance of early identification and management to improve maternal and fetal outcomes. Women with known risk factors, particularly those with a history of multiple cesarean sections, should undergo thorough antenatal screening for PAS, regardless of the placental site. Comprehensive management strategies tailored to the individual patient's needs are essential to mitigate the severe complications associated with these disorders [14].

## Case presentation

A 25-year-old multiparous woman with gravida2 parity1 living1 (G2P1L1) at 36 weeks of gestation, with a history of cesarean section performed 16 months prior for chorioamnionitis, presented to the labor and delivery unit experiencing active labor pains. Antenatal ultrasonography revealed the placenta implanted on the posterior surface of the upper uterine segment. Given the short inter-delivery interval, an emergency preterm lower segment cesarean section (LSCS) with pfannenstiel incision under spinal anesthesia, further converted to general anesthesia, was indicated and subsequently performed.

**Figure 1:**
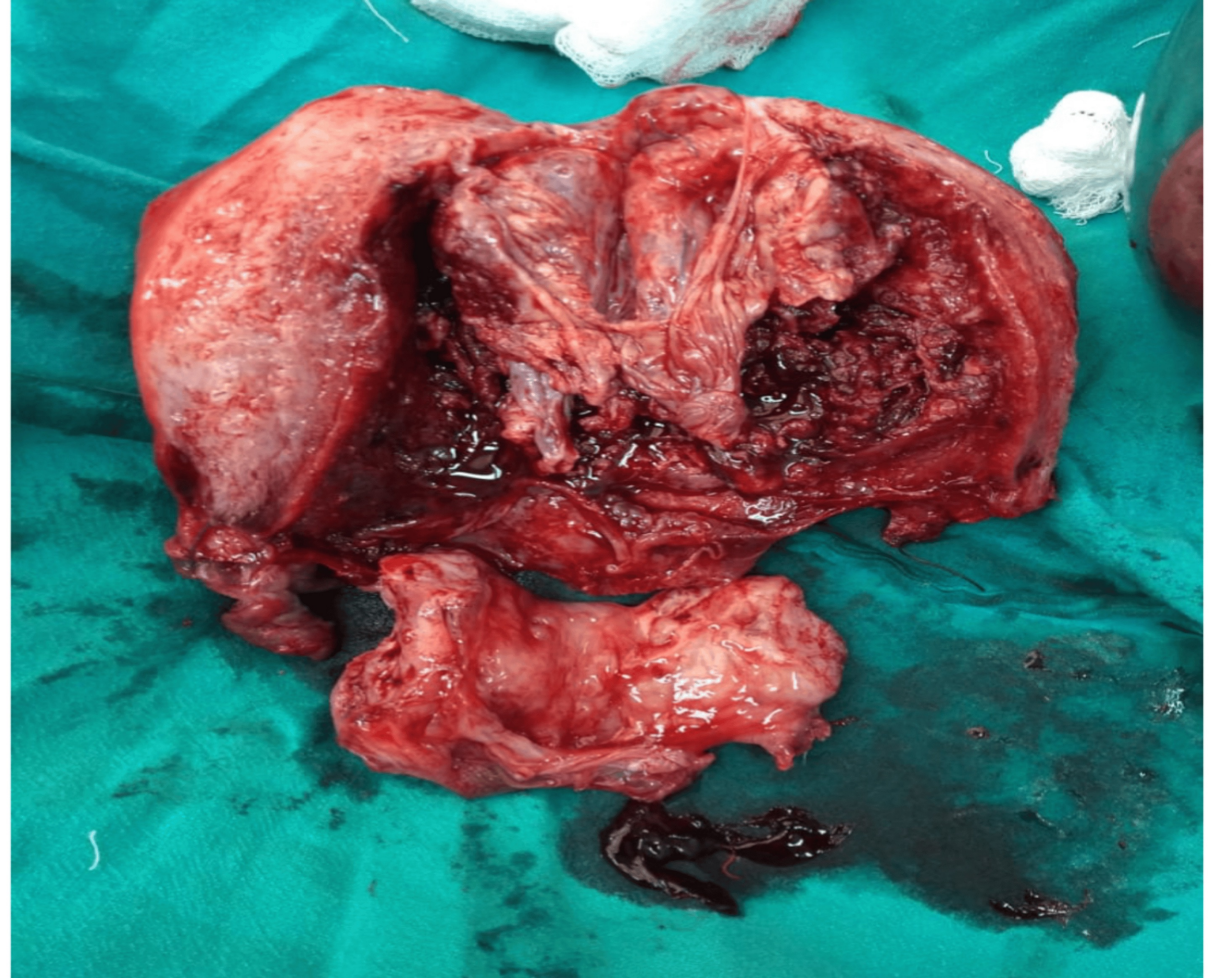
Intraoperatively, the placenta was found over the fundus of the uterus on the posterior surface, which was morbidly adherent.

Intraoperatively, the placenta was found to be morbidly adherent to the fundus of the uterus (Figure 1), consistent with a diagnosis of placenta increta. Following a thorough discussion with the patient and her family regarding expectant and surgical management options, an emergency obstetric total hysterectomy was conducted to effectively manage the placenta accreta syndrome (PAS). Intraoperative management included the transfusion of one unit of packed cell volume (PCV).

Postoperatively, the patient required additional transfusions of two units of PCV and two units of fresh frozen plasma (FFP). On the third postoperative day, she developed right lung consolidation, necessitating admission to the Obstetric Intensive Care Unit (OBICU) for five days for intensive monitoring and treatment. The remainder of the postoperative period was uneventful, and the patient was discharged on the 10th postoperative day in stable condition, along with her healthy newborn, a female infant weighing 1.8 kg.

**Figure 2:**
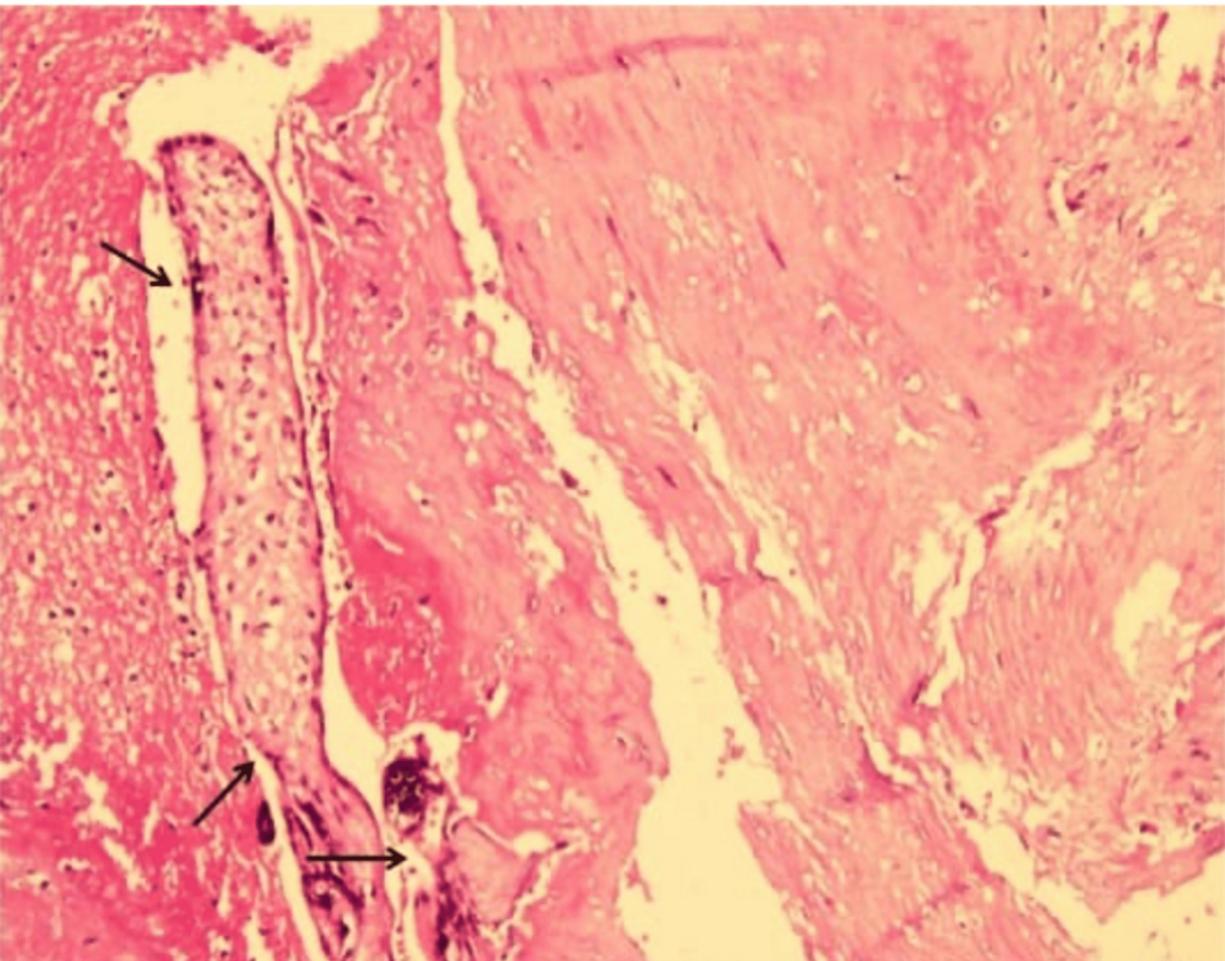
The section from endometrium shows the presence of chorionic villi invading myometrium without intervening decidua, suggestive of placenta increta. Myometrium shows the hypertrophy of the muscle.

Histopathological examination of the resected uterus confirmed the diagnosis of placenta increta (Figure 2). Sections from the endometrium demonstrated the presence of chorionic villi invading the myometrium without intervening decidua, and hypertrophy of the myometrial muscle was observed. This case underscores the critical importance of early diagnosis and timely surgical intervention in managing morbidly adherent placentation to mitigate the associated maternal and fetal risks.

## Discussion

Placenta increta, a severe variant of the placenta accreta spectrum (PAS), presents a significant challenge in obstetric management due to its deep invasion into the myometrium without reaching the uterine serosa [1]. The condition's increasing incidence is linked to the rising rates of cesarean sections and advanced maternal age, necessitating improved diagnostic and management strategies [15].

The patient's history of a cesarean section performed 16 months prior was a significant risk factor for placenta increta. The risk of PAS disorders escalates with the number of prior cesarean sections, with studies indicating a risk increase from 3% with one previous cesarean to 67% with four or more [6]. Other contributing factors include placenta previa, previous uterine surgeries such as dilation and curettage or myomectomy, high parity, and the use of assisted reproductive technologies [7,16]. These risk factors highlight the need for vigilant prenatal care and consideration of PAS in patients with such histories.

The management of PAS, particularly placenta increta, often necessitates a multidisciplinary approach. In this case, an emergency obstetric total hysterectomy was performed following intraoperative identification of a morbidly adherent placenta over the uterine fundus. This approach is supported by current guidelines, which recommend cesarean hysterectomy as the definitive treatment to prevent life-threatening hemorrhage [12,17]. The importance of a prepared and coordinated surgical team cannot be overstated, as prompt action and adequate intraoperative support are crucial for patient outcomes.

Intraoperative and postoperative management focuses on controlling hemorrhage and maintaining hemodynamic stability. The patient in this case required intraoperative and postoperative transfusions of packed cell volume (PCV) and fresh frozen plasma (FFP), highlighting the need for readily available blood products and a well-structured transfusion protocol [18]. The development of right lung consolidation postoperatively underscores the necessity for vigilant monitoring for potential complications, including respiratory issues, which are common in patients undergoing extensive surgical procedures [5].

Histopathological examination is essential for confirming the diagnosis of placenta increta. The presence of chorionic villi invading the myometrium without intervening decidua, along with hypertrophy of the myometrial muscle, confirms the diagnosis. These histological findings are critical for guiding future management, particularly in advising patients about risks in subsequent pregnancies [19].

Recent advancements in the management of PAS include interventional radiology techniques, such as preoperative arterial catheterization, to occlude pelvic blood flow and reduce intraoperative bleeding. However, the effectiveness and safety of these techniques require further research and the development of standardized protocols.

This case highlights several critical considerations in managing PAS disorders. First, early and accurate antenatal diagnosis is crucial for planning appropriate management strategies and reducing the need for emergent interventions. Second, a multidisciplinary approach involving obstetricians, anesthesiologists, and interventional radiologists is essential for comprehensive care. Third, patient education and counseling are vital, particularly for women with known risk factors, to ensure they understand the potential risks and the importance of regular antenatal monitoring.

In summary, placenta increta, as part of the PAS disorder, presents significant challenges in obstetric care. The increasing incidence necessitates heightened awareness and improved diagnostic and management strategies. Early diagnosis through advanced imaging, coupled with a multidisciplinary management approach, is critical in mitigating the severe maternal and fetal complications associated with PAS disorders. Further research is needed to refine diagnostic criteria and develop standardized management protocols to improve outcomes for affected women [18,20].

## Conclusions

Placenta increta, as a component of the placenta accreta spectrum (PAS) disorders, presents significant challenges in obstetric care due to its association with severe maternal morbidity and potential mortality. This case highlights the critical importance of early and accurate antenatal diagnosis through advanced imaging techniques such as ultrasound and MRI, which facilitate meticulous planning and timely intervention. The management of PAS, particularly placenta increta, often necessitates a multidisciplinary approach, involving obstetricians, anesthesiologists, and interventional radiologists to address the complexities of the condition effectively. Emergency obstetric hysterectomy remains the cornerstone of treatment to prevent life-threatening hemorrhage. Adequate intraoperative and postoperative care, including blood transfusion protocols and vigilant monitoring for complications, is essential to ensure favorable outcomes. This case underscores the necessity for heightened awareness, improved diagnostic strategies, and comprehensive management protocols to mitigate the risks associated with PAS disorders. Further research and development of standardized guidelines are imperative to enhance the care and outcomes for affected women, ensuring that those at risk receive appropriate antenatal surveillance and intervention.
